# Segmental Spinal Dysgenesis

**DOI:** 10.31486/toj.19.0094

**Published:** 2020

**Authors:** Emily Knafl, Neil U. Lall, Miles Love, Cuong Bui, Andrew J. Steven

**Affiliations:** ^1^ The University of Queensland Faculty of Medicine, Ochsner Clinical School, New Orleans, LA; ^2^ Department of Radiology, Emory University School of Medicine, Atlanta, GA; ^3^ Department of Radiology, Ochsner Clinic Foundation, New Orleans, LA; ^4^ Department of Neurosurgery, Ochsner Clinic Foundation, New Orleans, LA

## INTRODUCTION

Segmental spinal dysgenesis (SSD) is a rare congenital deformity that occurs when portions of the spine and spinal cord fail to develop properly.^1^ SSD was originally defined as agenesis or dysgenesis localized to the lumbar or thoracolumbar regions of the spine,^2^ but the definition was later expanded to congenital spinal dysraphism that must include (1) paraparesis/paraplegia, including lower limb aberrations; (2) more than one segment vertebral abnormality which may include kyphosis or kyphoscoliosis; (3) the absence or malformation of a portion of the spinal cord, along with associated nerve roots anywhere from the cervical spine to the sacrum; and (4) the presence of spinal cord distal to the affected region of cord.^3^

Dysraphic conditions such as SSD have been attributed to a mishap in the development of the early neural tube that results in a nonclosure.^2,4,5^ The diversity of accompanying malformations in other organs is thought to be the result of a singular event in embryogenesis that simultaneously disrupts all 3 embryonic germ layers.^4,5^ Gastrulation is hypothesized to be the pivotal moment when the germ layers are in enough proximity to allow the derangement to occur.^2,4,5^ During this phase, tissues migrate to form the trilaminar disk with the ectoderm, mesoderm, and endoderm superimposed together, and these layers initiate the early development of the neural tube.^2,4,5^ Malformations of other organs that are often associated with spinal dysraphism include hindgut duplication, horseshoe kidney, dextrocardia, and imperforate anus.^4,5^

We describe a rare case of SSD and discuss features of imaging, including aspects of prenatal imaging.

## HISTORY

During a routine prenatal ultrasound at 30 weeks and 2 days of gestation of a gravida 1 parity 0 patient with no significant medical history, limb anomalies were identified in a female fetus. In particular, the femur length and radius measurements were behind anticipated measurements for gestational age by 3-4 and 5 weeks, respectively. Follow-up fetal magnetic resonance imaging (MRI) performed at 33 weeks 0 days of gestation confirmed that the lower extremities were short and curved with clubfoot deformities.

Upon delivery, the infant had visible lower extremity deformities with legs fixed in a crossed position. The legs could not be straightened and lacked palpable pulses, and the left ankle had mild discoloration. The patient exhibited bilateral clubfoot deformities, atrophied calf and feet muscles, and an unusual spinal contour deformity. Follow-up with spinal ultrasound, MRI, and computed tomography (CT) with 3-dimensional (3-D) reconstruction was arranged.

## RADIOGRAPHIC APPEARANCE

### Prenatal Imaging

**Figure 1.**
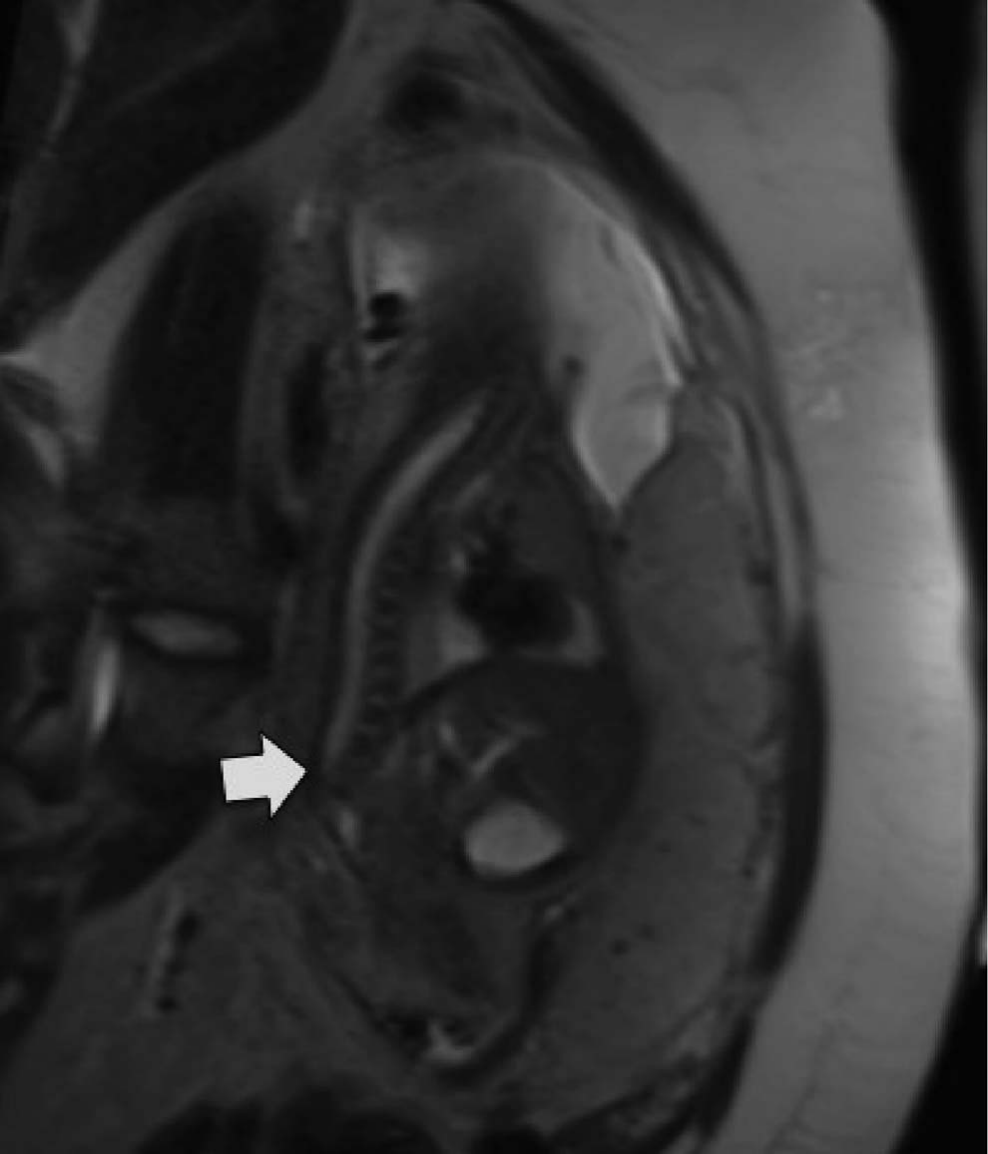
**Fetal magnetic resonance imaging. T2-weighted HASTE (half-Fourier single-shot turbo spin-echo) oblique reconstruction in the sagittal plane of the thoracolumbar and sacral spine shows a decreased number of lumbar vertebrae and a diminutive sacrum (arrow).**

Fetal MRI revealed a decreased number of lumbar vertebrae, a diminutive sacrum, and lumbar deformity (Figure 1). Additional lower extremity abnormalities included short femurs and clubfoot deformity.

### Postnatal Imaging

**Figure 2.**
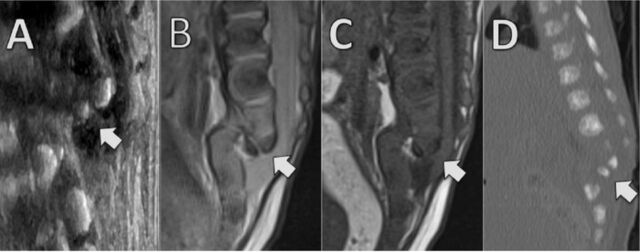
**Postnatal spinal imaging: (A) sagittal ultrasound, (B) sagittal T2-weighted magnetic resonance imaging (MRI), (C) sagittal T1-weighted MRI, and (D) sagittal reconstruction of a noncontrast computed tomography scan. Images show marked lumbar spine abnormalities; vertebral body hypoplasia; absent posterior segments in L3-L5; anterior displacement of the sacrum relative to the upper lumbar spine, resulting in a focal z-shaped deformity and kyphosis; diminutive sacrum; and a low-lying, posteriorly tethered spinal cord adherent to the dura with distorted morphology. The arrows across all images indicate the z-shaped deformity and also highlight where the tethered cord is most evident in magnetic resonance images B and C.**

Postnatal spinal evaluation with ultrasound showed markedly deformed lumbar vertebrae with a focal kyphosis and anterior translation of the lower lumbar vertebral segments, creating a z-shaped configuration (Figure 2A). Cord tethering was also noted, with the spinal cord extending below L2 to the level of the sacrum.

**Figure 3.**
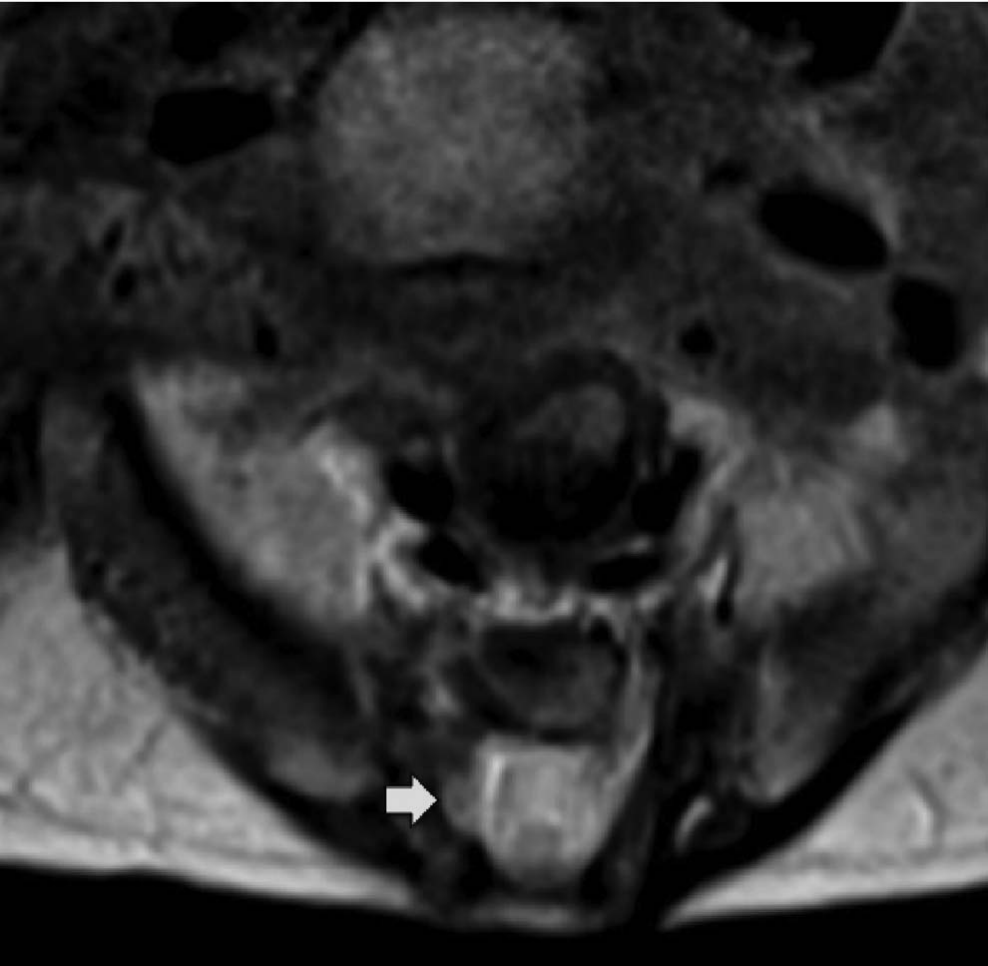
**Axial T2-weighted magnetic resonance imaging at the level of the sacrum shows the thecal sac with expansion and posterior cord tethering as a result of posterior spinal dysraphism (arrow).**

MRI (Figures 2B and 2C) and CT (Figure 2D) performed for further anatomic assessment demonstrated that the cervical and thoracic vertebral bodies retained normal height, morphology, and alignment; however, marked deformity of the lumbar spine was revealed across all postnatal imaging modalities. The L1 vertebral body was hypoplastic with preservation of posterior elements, the L2 body was profoundly hypoplastic with diminutive posterior elements, and the L3-L5 vertebral segments were completely absent. An associated alignment abnormality at the lumbosacral junction with a 1-cm anterior displacement of the sacrum relative to the upper lumbar spine resulted in a focal z-shaped deformity and severe kyphosis (Figures 2B, 2C, and 2D). The sacrum was mildly diminutive in overall size, consistent with a component of caudal regression. Multilevel bony posterior spinal dysraphism was present, involving the remaining lumbar and sacral levels without open neural tube defect. Additionally, the spinal cord was low-lying and tethered posteriorly. Cord tethering was demonstrated best on MRI (Figures 2B and 2C). Expansion of the thecal sac because of posterior spinal dysraphism was evident on axial T2-weighted MRI of the sacrum (Figure 3).

**Figure 4.**
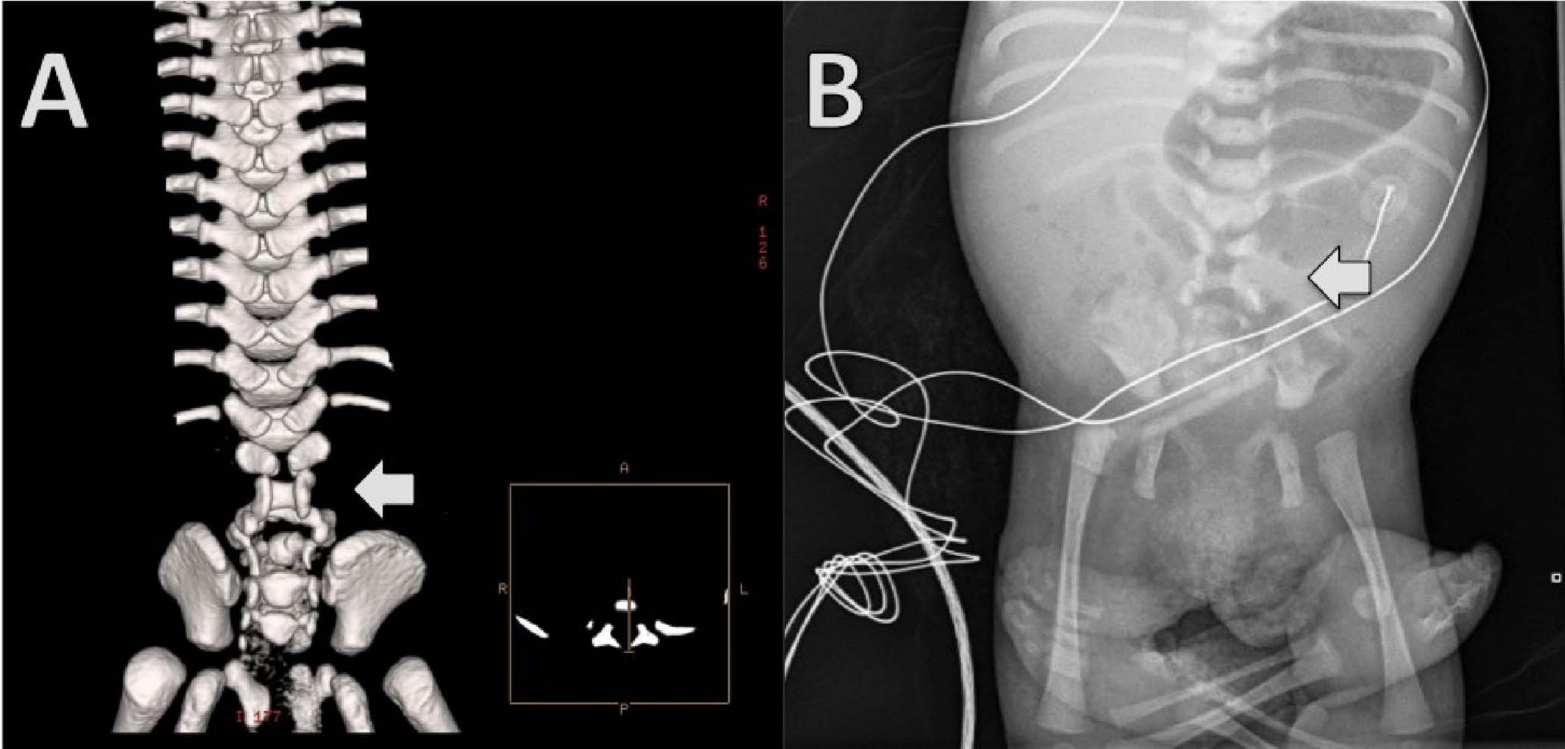
**(A) Computed tomography 3-dimensional spine reconstruction and (B) chest and abdominal x-ray show multilevel segmental spinal dysraphism involving the lumbar and sacral levels with hypoplastic vertebral elements. The most affected region of lumbar spine is indicated by arrows.**

A 3-D reconstruction of the noncontrast CT image (Figure 4A) and chest and abdominal x-ray (Figure 4B) allowed better delineation of the patient's overall abnormal bony anatomy.

Associated extraspinal structural abnormalities were ruled out with follow-up imaging; no congenital malformations were shown on renal ultrasound and no Chiari malformation on brain MRI. However, hip radiographs revealed bilateral acetabular dysplasia with hip dislocation.

## DISCUSSION

### Imaging

Postnatally, MRI is well established as the imaging modality of choice for evaluation of spinal defects such as SSD.^2^ MRI is best able to visualize soft tissue, cerebrospinal fluid, and neural structures within the affected region.^6^ CT has a complementary role and provides optimal delineation of bony defects, while 3-D reconstructions can provide enhanced visualization of the overall deformity and can be invaluable in planning interventions.^6^

As per the diagnostic requirements, characteristic imaging findings include a localized deformity of the spine with associated scoliosis/kyphosis and abnormality of the underlying cord/nerve roots, with continuation of the spinal cord distal to the affected region, typically with a thickened and low-lying configuration.^2^ The spinal canal may be severely narrowed or even interrupted at the gibbus apex of the kyphotic deformity, with associated hypoplasia or absence of the cord traversing this region.^2^ The appearance can be widely variable, depending on the level and severity of the deformity and the potential presence of associated closed spinal dysraphisms.^2^

### Prenatal Diagnosis of Segmental Spinal Dysgenesis

Prenatal imaging cases of SSD are scarce, with published cases primarily involving ultrasound^7-9^ and MRI.^5,10^ Similar to previously published examples of SSD, the lower extremity abnormalities in our case were also the first findings identified via routine ultrasound screening. Subsequent fetal MRI confirmed clubfoot deformities and a spinal abnormality, but the spinal deformity was much better characterized on postnatal imaging. Fetal MRI is associated with several challenges such as patient motion. Unlike imaging a newborn, fetal MRI offers little to no opportunity to reduce patient motion via swaddle techniques. Therefore, only a limited number of fast-acquisition techniques can be used, and these sequences trade spatial resolution and/or contrast resolution for temporal resolution, thus limiting the quality of anatomic evaluation. Further, even these images are often partially degraded by motion, resulting in blurring or gaps in coverage. For example, in our case, fetal motion prevented image acquisition in good alignment with the spinal canal, and the quality of the examination was further degraded by a large maternal body habitus. Multiplanar reconstructions created from the acquired off-axis images allowed for a true sagittal image capable of delineating the spinal truncation and malalignment. While informative and timely, fetal MRI often does not preclude further imaging after birth in the management of complex spine cases.

When diagnosing SSD, an important consideration is alternative spinal abnormalities such as multiple vertebral segmentation disorder, congenital vertebral displacement, and caudal regression syndrome.^2^ Even though the lesions in closed spinal dysraphisms are covered with skin, some cases may have cutaneous and soft-tissue manifestations such as dorsal dimples, dermal sinuses, lumbar skin tags, or localized hirsutism that can provide additional clinical clues.^11^ Lower extremity deformities and anorectal malformations are other indications that further imaging is warranted.^11^ Associated spinal cord abnormalities (diplomyelia, diastematomyelia, dorsal dermal sinus, neuroenteric fistula, terminal myelocystocele, spina bifida) and vertebral abnormalities (hemivertebrae, block laminae, butterfly vertebrae) may occur concomitantly, and patients should be evaluated for these conditions.^2^

### Management Strategies

Therapeutic management of SSD requires approaching each patient individually, depending on where the patient's deformities fall along the broad spectrum of severity. Complications may include cord tethering, resulting in progressive neural injury and instability of the spine leading to gradual kyphosis—both of which can threaten any residual function within the spinal cord.^1^ Surgical treatment options are considered on an individual basis and depend on neurologic function, spinal stability, pain, and the patient's age and size. Because these cases generally involve closed (skin covered) dysraphism, the need to surgically intervene during the neonatal period is rare. Patients should be treated at a tertiary care center, ideally by a multidisciplinary spinal dysraphism care team (eg, a spina bifida clinic), where patients can be followed and treated by pediatricians and subspecialists in pediatric neurosurgery, pediatric orthopedics, pediatric physical medicine and rehabilitation, and pediatric urology. If needed, interventions are generally performed for spinal untethering and/or spinal deformity correction and instrumented fusion stabilization.

Serial leg bracing was performed for our patient, resulting in lessened deformity but persistent poor functionality. The patient had active hip abduction and flexion but no functional motor activity below the hips. Abnormal sensation below the hips was also evident during examination. No signs of Chiari malformation or hydrocephalus were noted, and because of a lack of function to preserve, no neurosurgical interventions were performed.

## CONCLUSION

This case highlights a rare and severe example of spinal dysraphism, SSD. Patients with this condition should be treated at a tertiary care center, ideally by a multidisciplinary spinal dysraphism care team.

## Notes

This article meets the Accreditation Council for Graduate Medical Education and the American Board of Medical Specialties Maintenance of Certification competencies for Patient Care and Medical Knowledge.

## References

[1] Scott RM, Wolpert SM, Bartoshesky LE, Zimbler S, Karlin L Segmental spinal dysgenesis. Neurosurgery. 1988 4;22(4):739-744. doi: 10.1227/00006123-198804000-00021. 3374785

[2] Tortori-Donati P, Fondelli MP, Rossi A, Raybaud CA, Cama A, Capra V Segmental spinal dysgenesis: neuroradiologic findings with clinical and embryologic correlation. AJNR Am J Neuroradiol. 1999 3;20(3):445-456. 10219410 PMC7056058

[3] Chellathurai A, Ayyamperumal B, Thirumaran R, Kathirvelu G, Muthaiyan P, Kannappan S Segmental spinal dysgenesis – “redefined.” Asian Spine J. 2019 4;13(2):189-197. doi: 10.31616/asj.2018.0076. 30472824 PMC6454287

[4] Dias MS, Walker ML The embryogenesis of complex dysraphic malformations: a disorder of gastrulation? Pediatr Neurosurg. 1992;18(5-6):229-253. doi: 10.1159/000120670. 1476931

[5] Valdez Quintana M, Michaud J, El-Chaar D, El Demellawy D, Nikkel SM, Miller E Fetal segmental spinal dysgenesis and unusual segmental agenesis of the anterior spinal artery. Childs Nerv Syst. 2016 8;32(8):1537-1541. doi: 10.1007/s00381-016-3054-x. 26969176

[6] Bristol RE, Theodore N, Rekate HL Segmental spinal dysgenesis: report of four cases and proposed management strategy. Childs Nerv Sys. 2007 3;23(3):359-364. doi: 10.1007/s00381-006-0228-y. 17021723

[7] Fratelli N, Rich P, Jeffrey I, Bahamaie A, Thilaganathan B, Papageorghiou AT Prenatal diagnosis of segmental spinal dysgenesis. Prenat Diagn. 2007 10;27(10):979-981. doi: 10.1002/pd.1807. 17611945

[8] Selvakumaran S, Berger H, Sgro M, Zipori Y Atypical segmental spinal dysgenesis: prenatal diagnosis with postpartum short-term follow-up. J Obstet Gynaecol Can. 2019 12;41(12):1695. doi: 10.1016/j.jogc.2018.07.012. 30773310

[9] Hughes L, McCarthy R, Glasier C Segmental spinal dysgenesis: a report of three cases. J Pediatr Orthop. 1998 Mar-Apr;18(2):227-232. 9531407

[10] von Koch CS, Glenn OA, Goldwtein RB, Barkovich AJ Fetal magnetic resonance imaging enhances detection of spinal cord anomalies in patients with sonographically detected bony anomalies of the spine. J Ultrasound Med. 2005 6;24(6):781-789. doi: 10.7863/jum.2005.24.6.781. 15914682

[11] Schwartz E, Rossi A Congenital spine anomalies: the closed spinal dysraphisms. Pediatr Radiol. 2015 9;45 Suppl 3:S413-S4139. doi: 10.1007/s00247-015-3425-6. 26346147

